# Highly aqueously stable C_60_‐polymer nanoparticles with excellent photodynamic property for potential cancer treatment

**DOI:** 10.1002/SMMD.20230033

**Published:** 2023-12-20

**Authors:** Dan Wang, Jianyang Zhao, Roger J. Mulder, Julian Ratcliffe, Chunru Wang, Bo Wu, Jinquan Wang, Xiaojuan Hao

**Affiliations:** ^1^ Guangdong Pharmaceutical University Guangzhou Guangdong China; ^2^ Manufacturing Commonwealth Scientific and Industrial Research Organisation (CSIRO) Clayton Victoria Australia; ^3^ Beijing National Laboratory for Molecular Sciences Institute of Chemistry Chinese Academy of Sciences Beijing China; ^4^ Joint Research Centre on Medicine The Affiliated Xiangshan Hospital of Wenzhou Medical University Ningbo Zhejiang China; ^5^ Zhejiang Engineering Research Centre for Tissue Repair Materials Wenzhou Institute University of Chinese Academy of Sciences Wenzhou Zhejiang China

**Keywords:** C_60_ fullerene, C_60_‐polymer nanoparticles, cancer treatment, photodynamic therapy

## Abstract

Fullerenes are a class of carbon nanomaterials that find a wide range of applications in biomedical fields, especially for photodynamic cancer therapy because of its photosensitive effect. However, hydrophobic fullerenes can only be dispersed in organic solvents which hinders their biomedical applications. Here, we report a facile method to prepare highly water‐dispersible fullerene (C_60_)‐polymer nanoparticles with hydrodynamic sizes of 50–70 nm. Hydrophilic random copolymers containing different ratios of polyethylene glycol methyl ether methacrylate and 2‐aminoethylmethacrylamide were synthesized for conjugating with C_60_ molecules through efficient nucleophilic Michael addition reaction between amine groups from hydrophilic polymer and carbon‐carbon double bonds from C_60_. As a result, the amphiphilic C_60_‐polymer conjugates could be well dispersed and nano‐assembled in water with a C_60_ concentration as high as 7.8 mg/mL, demonstrating a significant improvement for the solubility of C_60_ in an aqueous system. Owing to the high C_60_ content, the C_60_‐polymer nanoparticles showed a strong photodynamic therapy effect on human lung cancer cells (A549) under light irradiation (450 nm) in both 2D cell culture and 3D spheroid culture, while demonstrating ignorable cytotoxicity under dark. This highly efficient and convenient method to prepare water‐dispersible C_60_‐polymer conjugates may have a great impact on the future biomedical applications of fullerenes.

## Graphical abstract

This work used controlled living free radical polymerization (reversible addition‐fragmentation chain transfer) to prepare copolymers of precise compositions. The conjugation method of C_60_ molecules with polymers is more efficient and simpler to generate highly water‐dispersible nanoparticles compared to literature methods. In addition, the C_60_ concentration of this work is much higher than literature reported, yet the stability of nanoparticle aqueous solution is superior, which can be stored at room temperature for more than 1 year without aggregation. Such highly aqueously stable C_60_ nanoparticles showed non‐cytotoxicity under dark conditions but high photo‐cytotoxicity with light irradiation (visible light, short time and low intensity), which can lead to targeted killing of cancer cells.

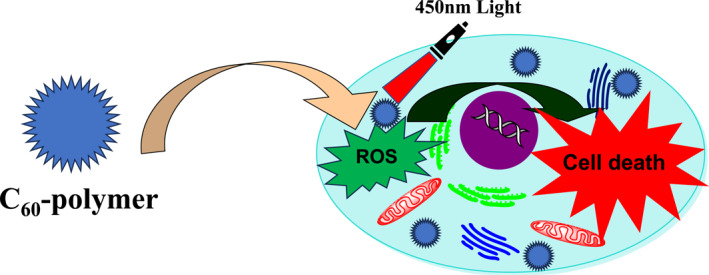

**Key points**

- This work used controlled living free radical polymerization (reversible addition‐fragmentation chain transfer, RAFT) to prepare copolymers of precise compositions.
- The developed conjugation method of C_60_ molecules with water‐soluble RAFT polymers to generate highly water‐dispersible nanoparticles is more efficient and simpler compared to literature methods.
- In addition, the C_60_ concentration of this work is much higher than literature reported, yet the stability of nanoparticle aqueous solution is superior, which could be stored at room temperature for more than 1 year without aggregation.
- Such highly aqueously stable C_60_ nanoparticles showed non‐cytotoxicity under dark conditions but high photo‐cytotoxicity with light irradiation (visible light, short time and low intensity), which can lead to targeted killing of cancer cells.

## 1 INTRODUCTION

Fullerenes and their derivatives are very attractive due to their various promising biomedical applications such as anti‐viruses,^1^, ^2^, ^3^, ^4^, ^5^ antioxidant, neuroprotective, antimicrobial, and photodynamic therapy (PDT), which is attributed to their versatile physicochemical properties.^6^, ^7^, ^8^, ^9^, ^10^, ^11^, ^12^ Among those in particular, owing to the photo‐sensitive property, fullerene was recently used in PDT as a novel photosensitizer, which is an efficient technology with highly selective cytotoxicity against cancer cells in response to light, showing great prospects in fighting against cancers.^10^, ^13^, ^14^, ^15^ To better realize such applications it is necessary to make the water‐insoluble fullerenes^16^, ^17^ well disperse in the aqueous environment. Over the past 30 years, great efforts have been committed to make fullerene dispersible in water.^18^, ^19^ Solubilizing agents, such as cyclodextrin, polyvinylpyrrolidone, calixarene, and liposome, were used to wrap fullerenes and make them soluble in water.^20^, ^21^, ^22^, ^23^, ^24^, ^25^ A variety of hydrophilic and biomolecular units, such as hydroxyl,^26^, ^27^, ^28^ carboxyl,^29^, ^30^, ^31^ amino acid,^32^, ^33^, ^34^ peptide,^19^, ^35^, ^36^ and sugar,^2^, ^37^, ^38^ were conjugated to the surface of fullerene, resulting in many kinds of water‐soluble fullerene derivatives. Besides these, water‐soluble polymers conjugated to the fullerene surface,^39^, ^40^, ^41^ fullerene as the end cap of water‐soluble polymers,^42^, ^43^ fullerene connected to the side chain of water‐soluble polymers,^44^, ^45^ main chain water‐soluble fullerene‐polymers,^46^ copolymerization of fullerene functionalized monomer with *N*‐vinylpyrrolidone^47^ are also useful methods to achieve water‐soluble targets.^48^ Despite considerable progresses, there is still a long distance toward its real applications. The main reason is the difficulty of achieving a high‐concentration fullerene water solution with long‐term stability. Therefore, an efficient method for preparing a high‐concentration fullerene aqueous solution is highly needed.

Poly(ethylene glycol) (PEG) is a non‐toxic, synthetic, water‐soluble polymer of great interest in bio‐applications. In 1977, Davis and Abuchowski connected this polymer to bovine serum albumin and liver catalase protein. They found that the polymer increased the systemic circulation time in the body and decreased the immunogenicity of the protein without significantly compromising activity.^49^ In 1990, FDA approved the first PEGylated drug for severe combined immunodeficiency disease.^50^ PEG has been extensively studied and used in biomedical applications as it is well known to improve drug solubility, decrease immunogenicity, increase stability and retention time in blood, reduce proteolysis and excretion, and allow reduced dosing frequency.^51^ When attaching PEG to nanoparticles, it protects the nanoparticles from aggregation, opsonization, and phagocytosis, thereby prolonging circulation time.^52^ With a great interest to this polymer, more sophisticated graft copolymers with PEG side chains were developed.^53^, ^54^ The nonlinear comb‐shaped analogs not only keep the unique properties of linear PEG, but also possess the advantage of introducing various functional groups during the synthesis, resulting in many new applications such as hydrogel,^55^ stimuli polymer,^56^ and lithium ion battery.^57^

In this study, a completely new, simple, and efficient method was developed to prepare highly water‐dispersible fullerene‐polymer nanoparticles by conjugating C_60_ to a water‐soluble copolymer. Firstly, nonlinear random copolymers containing polyethylene glycol methyl ether methacrylate (PEGMA) and 2‐aminoethylmethacrylamide (AEMA) of different compositions were designed and synthesized by reversible addition‐fragmentation chain transfer (RAFT) polymerization,^58^, ^59^ a very important technology in polymer synthesis field, which can well control the polymerization process, polydispersity of molecular weight, and structure of polymers. C_60_ fullerene molecules were then conjugated to the polymer side chains by amine addition reaction between AEMA and C_60_ fullerene. It is expected that the water‐soluble PEG side chains around the fullerene molecules would wrap them and create an aqueous microenvironment, thus making them highly water dispersible with excellent stability. The as‐prepared C_60_‐polymer aqueous solution was evaluated toward human lung cancer cells (A549) for its potential application in PDT.

## 2 MATERIALS AND METHODS

### 2.1 Materials and analytical techniques

PEGMA (*M*_*n*_ = 500), ethylenediamine, HCl, 2‐propanol, methacrylic anhydride, acetone, dioxane, *N*,*N*‐dimethyl formamide (DMF), cyclohexane, and 4‐cyano‐4‐(phenylcarbonothioylthio) pentanoic acid (RAFT agent) were purchased from Sigma‐Aldrich (Australia). 4,4′‐Azobis(4‐cyanovaleric acid) (ACVA) was purchased from MP Biomedicals, which was recrystallized in methanol twice before use. C_60_ was kindly provided by Xiamen Funano New Material Technology Company and used as received. 1,8‐Diazabicyclo[5.4.0]undec‐7‐ene (DBU) was purchased from Alfa Aesar (Australia). The dialysis membrane (*M*_*w*_ cut‐off = 3500 Da) was purchased from Spectrum Laboratories Incorporated (USA). All chemicals were used as received otherwise stated. AEMA monomer was synthesized in house as previously reported.^60^

^1^H NMR and ^13^C NMR spectra were acquired on Bruker Av400 and Av500 NMR spectrometers; and the obtained spectra were processed using Topspin 3.5 NMR software. Gel permeation chromatography (GPC) measurements were conducted at 80°C on a Shimadzu HPLC system using a PL 5.0 mm bead‐size guard column and four linear PL (Styragel) columns with refractive index detection. *N*,*N*‐dimethyl acetamide containing lithium bromide (2.1 g/L) was used as the mobile phase (flowrate: 1 mL/min). The calibration was performed using near‐monodisperse poly(methyl methacrylate) standards from Polymer Laboratories. UV‐Vis spectra were recorded on a Varian 50 Bio spectrophotometer. IR spectra were recorded on a Nicolet 6700 FTIR spectrometer (Thermo ScientificTM) with a diamond accessory. Mass spectra of polymer and polymer conjugates were collected on a Bruker Ultraflextreme mass spectrometer using Laser Desorption Ionization (no matrix employed). Electrospray ionization mass spectra were recorded on a liquid chromatography mass spectrum system (Thermo ScientificTM Q ExactiveTM Hybrid Quadrupole‐Orbitrap Mass Spectrometer). The morphologies of the fullerene‐polymer nanoparticles in water were observed using cryogenic transmission electron microscopy. Firstly, copper grids (200‐mesh) coated with carbon film were glow discharged in nitrogen to make them hydrophilic. Then, aliquots of samples were dipped onto the grids and after 10 s adsorption, excess samples were removed from the grids by a filter paper. The grids were then plunged into liquid ethane cooled by liquid nitrogen. The samples were then placed onto a Gatan 626 cryoholder cooled by liquid nitrogen and examined by a Tecnai 12 transmission electron microscope (FEI) at 120 kV. Images were recorded using a FEI Eagle CCD camera. Dynamic light scattering (DLS) was used to measure the particle size and distribution of the fullerene‐polymer nanoparticles in water. The experiments were performed on a Malven Zetasizer (Nano‐ZS) equipped with a 4 mW HeNe Laser operating at 633 nm. The measurements were conducted at 25°C in standard disposable cuvettes and the scatter light was performed at an angle of 173°. Thermal gravimetric analysis (TGA) was conducted on a Mettler Toledo TGA2 star^e^ system by heating the samples from 25 to 1000°C at a rate of 15°C/min under a nitrogen atmosphere.

### 2.2 Synthesis of PEGMA‐AEMA random copolymers

A typical synthesis of a PEGMA‐AEMA random copolymer (polymer 1) is as follows. PEGMA (2.45 g, 4.90 mmol), AEMA (0.89 g, 5.40 mmol), RAFT agent (7.0 mg, 0.025 mmol), and ACVA (2.0 mg, 0.0071 mmol) were added to a vial equipped with a stirrer bar and a solvent mixture of dioxane (2 mL) and H_2_O (4 mL) was added to form a homogeneous solution. Then, the vial was sealed and deoxygenated by purging with high purity nitrogen gas for 30 min. The vial was then placed in an oil bath set at 70°C, kept stirred. The mixture was periodically sampled under nitrogen protection, and the conversion was measured by ^1^H NMR spectroscopy. When the conversion reached ∼50%, the mixture was transferred into a dialysis bag (*M*_*w*_ cut‐off = 3500 Da) and dialyzed against water at room temperature for 48 h. After dialysis, the solution was freeze‐dried. The obtained polymer was then characterized by ^1^H NMR spectroscopy and GPC. Polymers 2–7 were synthesized following a similar process except that the amounts of PEGMA and AEMA were adjusted according to the designed target degree of polymerization (DP).

### 2.3 Conjugation of C_60_ and copolymers

In a typical process, a mixture containing the as‐prepared polymer (0.30 g), C_60_ (0.15 g), DMF (9 mL), and DBU (1 mL) was prepared in a vial equipped with a stirrer bar, which was then sealed and deoxygenated by purging with purity nitrogen gas for 30 min. After that, the vial was placed in an oil bath set at 65°C and stirred for 6 h. Then, the mixture was kept stirring at room temperature for another 16 h. The mixture was then centrifuged and the upper solution was collected and dialyzed against water at room temperature for 48 h. Finally, the resultant solution from dialysis was characterized by UV‐Vis spectroscopy, MS, cryo‐TEM, and DLS, and the solid products obtained after freeze‐drying were characterized by IR, ^13^C NMR spectroscopy, and TGA.

### 2.4 Cell lines and cell culture

Human lung carcinoma cell line A549 was obtained from the cell bank of the Cell Institute of Sinica Academia. Cells were cultured in Dulbecco's Modified Eagle's Medium (Gibco) supplemented with fetal bovine serum (10%), penicillin (100 U/mL), and streptomycin (50 U/mL). All cells were maintained in a CO_2_ incubator (95% relative humidity, 5% CO_2_) at a constant temperature of 37°C.

### 2.5 Photocytotoxicity test on 2D A549 cells

The 3‐(4,5‐dimethylthiazol‐2‐yl)‐2,5‐diphenyltetrazolium bromide (MTT) assay was used to detect the cell viability as our previous report.^13^ Cells were seeded in a 96‐well plate and incubated for 24 h. Then the cells were divided into six groups: control group, control + light group, polymer group, polymer + light group, C_60_‐polymer conjugate group, and C_60_‐polymer conjugate + light group. Different concentrations of C_60_‐polymer conjugate or polymer were added and incubated for 4 h. The cells of the light group were irradiated for 5 min (450 nm, 12 J cm^−2^). Then, the medium was removed and replaced with fresh dulbecco's modification of eagle's medium (DMEM) medium (with 10% fetal bovine serum [FBS]). Next, all of the groups were incubated for additional 24 h. After that, the MTT solution (5 mg/mL in phosphate balanced solution [PBS]) was added to each well and incubated for 4 h. Then, the medium was replaced by dimethyl sulfoxide to dissolve blue‐violet crystals. The optical density (OD) of each well was measured on a scanner (Biorad) at a wavelength of 570 nm. Cell viability (%) = (OD treated − OD blank)/(OD control − OD blank) × 100%. The cell viability rate was set as 100% for the control group.

In addition, calcein am propidium Iodide (AM/PI) staining assay was conducted to detect the live/death viability of A549 cells.^13^ Cells were treated as described above, then the cells were washed twice with PBS. After that, the cells were incubated with Calcein AM/PI for 30 min at 37°C in a 5% CO_2_ incubator. Then, the cells were washed twice with PBS and were observed under an inverted fluorescence microscope (Zeiss Axio Observer D1).

### 2.6 Photocytotoxicity test on 3D A549 spheroids

A549 multicellular tumor spheroids (MCTSs) were generated using a method as our previous study.^13^ Briefly, 150 μL single‐cell suspension with 5000 cells was added to the flat‐bottom 96‐well plates pre‐coated with 0.5 mg agarose (in 50 μL DMEM). Then, the plates were incubated at 37ºC and 5% humidified CO_2_ incubator until spheroids formed. The photocytotoxicity experiments were performed when the average diameter of spheroids reached ∼400 μm after 3 daysʹ growth. MCTSs were divided into three groups, the first group was treated with C_60_‐polymer conjugate (100 μg/mL) but without light irradiation, the second group was treated with polymer (100 μg/mL) with light irradiation (450 nm, 12 J cm^−2^, 5 min), and the third group was treated with C_60_‐polymer conjugate (100 μg/mL) with light irradiation (450 nm, 12 J cm^−2^, 5 min). Then, the medium was replaced with fresh DMEM medium (with 10% FBS). The medium was changed every 2 days and MCTSs were measured using a microscope (Zeiss Axio Observer D1) to monitor the diameter of the spheroids. In addition, the live/death viability of A549 MCTSs was detected by the Calcein AM/PI staining assay as described above.

### 2.7 Light‐induced ^1^O_2_ generation in vitro

Electron paramagnetic resonance (EPR) spectroscopy (Bruker model A300 spectrometer) was applied to evaluate the ability of the C_60_‐polymer conjugate to generate singlet oxygen (^1^O_2_) under 450 nm light in the solution (PBS:methanol = 1:1). The trapping agent 2,2′,6,6′‐tetramethylpiperidine (TEMP) was used to monitor the reaction with C_60_‐polymer conjugate (100 μg/mL) and the generation of ^1^O_2_ using an irradiation power of 12 J cm^−2^ for 5 min, which yielded a characteristic EPR spectrum with an intensity ratio of 1:2:1. The group without irradiation served as the control.

The production of intracellular reactive oxygen species (ROS) ^1^O_2_ was detected by 2′,7′‐dichlorofluorescein diacetate (DCFH‐DA, Sigma−Aldrich) probe. A549 2D cultured cells were treated with C_60_‐polymer conjugate or polymer alone under dark or light as described above. After treatment, the cells were washed with PBS three times and incubated with DCFH‐DA (10 μM) for 30 min at 37°C in the dark. Then, the cells were washed twice with PBS to remove DCFH‐DA and were visualized by an inverted fluorescence microscope (Zeiss, Model Axio Observer D1). In addition, the procedure for investigating the production of intracellular ROS of 3D MCTSs was similar to that of A549 2D cells.

## 3 RESULTS

### 3.1 Synthesis of poly(PEGMA‐s‐AEMA) copolymers

**FIGURE 1:**
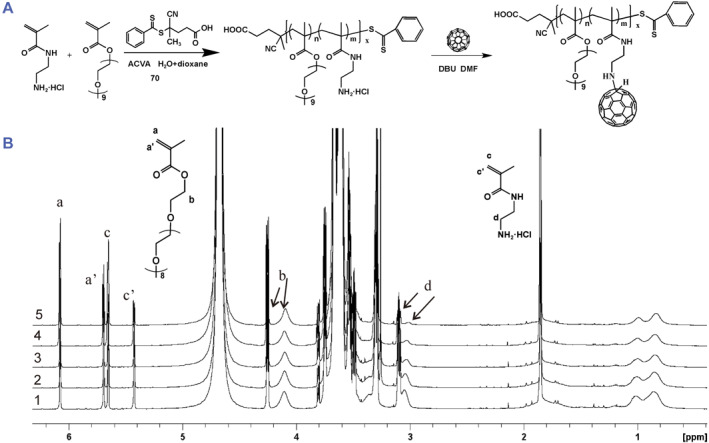
(A) Synthetic routes of poly(PEGMA‐s‐AEMA) and C_60_‐poly(PEGMA‐s‐AEMA) conjugate. (B) ^1^H NMR spectra of the reaction mixtures for conversion calculation (copolymers 1–5).

It is well known that the amine group can react with double bond of fullerene molecule with a high efficiency. RAFT technology was utilized to synthesize a series of random copolymers containing PEGMA and AEMA. PEGMA has side chains of PEG and was chosen as the co‐monomer to assist water solubility while AEMA contains an amine functional group and was chosen as co‐monomer for C_60_ attachment via amine addition. The synthetic route of the copolymer is shown in Figure 1A. In order to obtain a varied amount of fullerene conjugated to copolymers, random copolymers were designed with a fixed DP of PEGMA and varied DP of AEMA and synthesized using RAFT technology. In the experiments, the target DP of PEGMA was kept at 200 and target DP of AEMA was varied from 40 to 220. The conversions of PEGMA were controlled around 50%, resulting in copolymers with about 100 PEGMA repeating units and different number of AEMA repeating units (DP 14–101) (Table 1). Conversions of PEGMA were obtained by calculating the integral ratio of peak a to peak b and conversions of AEMA were obtained by calculating the integral ratio of peak cʹ to peak d (Figure 1B). The incorporation ratio of PEGMA is higher than that of AEMA in all cases attributed to different reactivity ratio between two monomers. For example, during the synthesis of polymer 1, the conversions of PEGMA and AEMA were 50 and 46 mol%, respectively, according to the ^1^H NMR analysis (Table 1), resulting in a random copolymer containing 100 repeating units of PEGMA and 101 repeating units of AEMA. The polydispersity index and number molecular weight *M*_*n*_ were measured by GPC and the results are also shown in Table 1.

**TABLE 1:** Synthesis conditions and characterization of the poly(PEGMA‐s‐AEMA) copolymers.

Random copolymer	Target DP [PEGMA]/[AEMA]	Conv (%)		DP by conversion^a^		*M*_*n*_ (GPC)^b^	PDI (GPC)^b^
		PEGMA	AEMA	PEGMA	AEMA		
1	200/220	50	46	100	101	43,250	1.23
2	200/176	50	43	100	76	35,710	1.21
3	200/133	51	39	102	52	40,150	1.33
4	200/88	55	40	110	35	37,660	1.20
5	200/40	54	36	108	14	44,690	1.24

Abbreviations: AEMA, 2‐aminoethylmethacrylamide; DP, degree of polymerization; GPC, gel permeation chromatography; PDI, polydispersity index; PEGMA, polyethylene glycol methyl ether methacrylate; PMMA, poly(methyl methacrylate).

^a^ DPs were calculated using conversion (DP = target DP × conv (%)).

^b^ PDI and number‐average molecular weight (*M*_*n*_) were determined by GPC using PMMA as the calibration standards.

### 3.2 Confirmation of fullerene conjugation with copolymers

**FIGURE 2:**
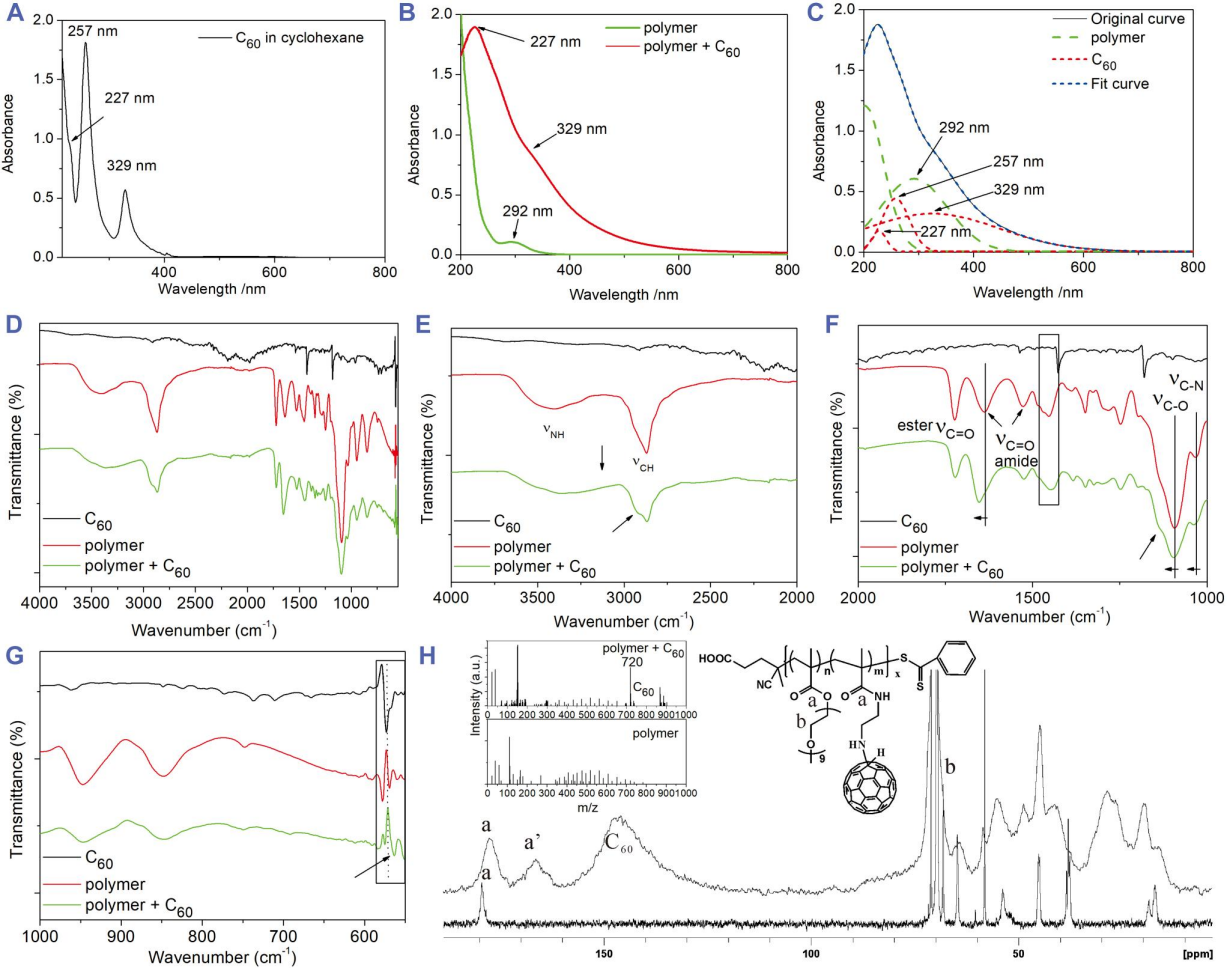
UV‐visible absorption spectra of (A) C_60_ in cyclohexane and (B) polymer and C_60_‐polymer conjugate in water; (C) Curve fitting of the C_60_‐polymer conjugate. IR absorption spectra of C_60_, polymer, and C_60_‐polymer conjugate: (D) 4000–550 cm^−1^, (E) 4000–2000 cm^−1^, (F) 2000–1000 cm^−1^, (G) 1000–550 cm^−1^. (H) ^13^C NMR spectra of polymer (bottom) acquired in D_2_O and C_60_‐polymer conjugate (upper) acquired in the solid state, inserted with MS spectra of polymer (bottom) and C_60_‐polymer conjugate (upper).

C_60_ was conjugated to the synthesized polymers according to the process depicted in Figure 1A. When fullerene is conjugated to the polymer it would be surrounded by the PEG side chains making the fullerene derivative water dispersible, thus being able to form stable nanoparticle solutions. The conjugates obtained by attaching C_60_ to copolymers 1–5 were extensively characterized by UV‐visible absorption, IR, NMR, cryo‐TEM, TGA, and MS, as discussed below. Figure 2A,B show UV‐visible absorption spectra of a C_60_‐polymer conjugate in comparison with pristine C_60_ and corresponding polymer. It can be observed from Figure 2A that pristine C_60_ dissolved in cyclohexane shows two characteristic peaks at 329 and 257 nm, and a shoulder peak at 227 nm, respectively, which are consistent with the previous report of C_60_ in tetrahydrofuran.^61^ Polymer itself shows an absorption peak around 292 nm and a strong absorption near 205 nm. The C_60_‐polymer conjugate in water shows a strong absorption tailing (400–600 nm) (Figure 2B), which is also consistent with the report.^61^ However, unexpectedly, the absorption curve shows a distinctive peak at 227 nm, which is coincidently the same wavelength of the shoulder peak of pristine C_60_. At the same time, the strong C_60_ peak at 257 nm does not show, and the C_60_ peak at 329 nm becomes weak. Curve fitting was conducted to determine the assignment of the absorption contributed by C_60_ and polymer, respectively. The three characteristic peaks of C_60_ (329, 257, and 227 nm) and two characteristic peaks of the polymer (205 and 292 nm) were chosen for the peak fitting analysis as shown in Figure 2C. The fitting curves (dotted lines) almost perfectly match the experimental absorption curves (solid line) with the peak positions and relative intensities consistent with expected values for C_60_ and polymer. The curve fitting result demonstrates that the absorption of C_60_‐polymer nanoparticle in water solution is indeed the combination of C_60_ and the as‐prepared polymer and the C_60_ structure was kept intact. On the other hand, the relative intensity of the peaks at 227 and 329 nm changed, indicating that the conjugation reaction occurred between the polymer and C_60_.

The typical IR spectra of pristine C_60_, polymer, and C_60_‐polymer conjugate are shown in Figure 2D–G, where some changes are observed. Firstly, after the conjugation, the polymer peak at 1453 cm^−1^ becomes broader (Figure 2F, rectangle mark), possibly due to the superposition of the pristine C_60_ peak (1426 cm^−1^) and polymer peak (1453 cm^−1^), respectively. In addition, the intensities of polymer peaks at 578 and 569 cm^−1^ have also changed (Figure 2G, rectangle mark) possibly due to the superposition of the pristine C_60_ peak (573 cm^−1^) and polymer peaks (578 and 569 cm^−1^), respectively. Secondly, the *ν*_C=O_ peak of amide shifts from 1636 to 1654 cm^−1^, the *ν*_C‐O_ peak shifts from 1092 to 1096 cm^−1^, the *ν*_C‐N_ peak shifts from 1031 to 1037 cm^−1^ (Figure 2F), respectively, demonstrating that the C_60_ molecules are covalently conjugated to the amine groups in the polymer. Thirdly, after conjugation, a new shoulder peak of *ν*_C‐H_ appears at 2920 cm^−1^ (Figure 2E), which can be attributed to *ν*_C‐H_ absorption near the C_60_; in addition, a new absorption appears around 3128 cm^−1^ (Figure 2E), probably attributed to the *ν* _= C‐H_ absorption of H‐C_60_. Such changes confirm that the amine addition reaction occurred. In summary, the IR results have demonstrated that the C_60_ molecules are covalently conjugated to the polymer side chains and both amine group and amide group participated in addition reaction.

In order to further confirm C_60_ fullerene is intact after conjugation with polymer, ^13^C NMR and MS experiments were performed. Figure 2H shows the ^13^C NMR spectrum of a typical as‐prepared polymer in D_2_O solution and the solid state ^13^C NMR spectrum of its corresponding C_60_‐polymer conjugate. The peak at 177.4 ppm (peak a) is the chemical shift from both ester and amide carbonyl carbons, which is split into two peaks after the conjugation reaction with C_60_ with an additional peak at 166.7 ppm (peak a’). This new peak at 166.7 ppm can be attributed to the amide carbonyl carbon where C_60_ is attached. When C_60_ is conjugated to the amine group of the AEMA unit, the chemical shift of the amide carbonyl carbon is changed due to the change of electronic microenvironment. This new peak provides evidence that C_60_ molecules are conjugated to the amine group. In addition, the peak at 146.8 ppm is the characteristic peak of C_60_ (quaternary fullerene carbons),^37^, ^44^ which demonstrates that the structure of C_60_ is intact. The mass spectra of the polymer and its corresponding C_60_‐polymer conjugate are shown in Figure 2H (inserted). It is clearly observed that C_60_ peak (m/z 720) appears in the spectrum of the C_60_‐polymer conjugate, which also demonstrates that the structure of C_60_ is intact. All the above results prove that C_60_ molecules are successfully conjugated to the polymer without destruction of C_60_ structure.

### 3.3 Morphology observation and C_60_ content determination

**FIGURE 3:**
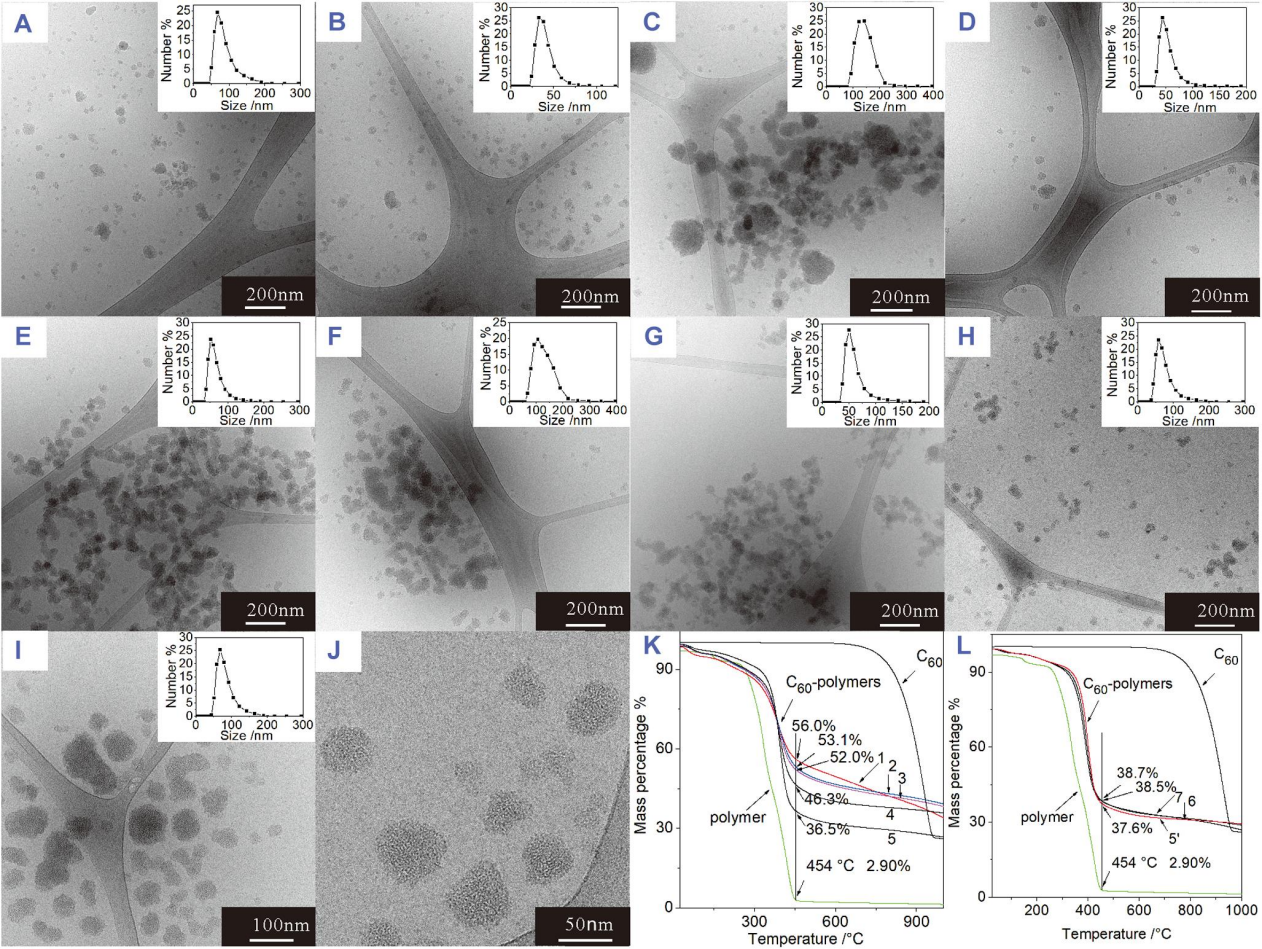
Cryo‐TEM images inserted with DLS curves of C_60_‐polymer conjugates. (A) C_60_‐polymer 1, (B) C_60_‐polymer 2, (C) C_60_‐polymer 3, (D) C_60_‐polymer 4, (E) C_60_‐polymer 5, (F) redispersed C_60_‐polymer 5, (G) C_60_‐polymer 6, (H) C_60_‐polymer 7, (I) C_60_ (high conjugation conc.)‐polymer 5, (J) higher magnification image of C_60_ (high conjugation conc.)‐polymer 5; TGA curves of C_60_, polymer, and C_60_‐polymer conjugates: (K) C_60_‐polymers 1–5 conjugates; (L) C_60_‐polymers 6–7 conjugates and C_60_ (high conjugation conc.)‐polymer 5 conjugate. DLS, dynamic light scattering; TGA, thermal gravimetric analysis.

Cryo‐TEM was used to examine the morphology of the C_60_‐polymer conjugates in water solution, avoiding the interference from conventional transmission electron microscopy (TEM) sample preparation. Figure 3A–J shows the cryo‐TEM images and DLS curves for different C_60_‐polymer conjugates. The C_60_ conjugates with polymer 1 and polymer 2 show a similar flake‐like morphology, with their particle peak positions of 67.9 and 33.2 nm, respectively, determined by DLS (Figure 3A,B). The decrease in size may be due to the decreased number of AEMA repeating units from 101 to 76, resulting in reduced number of C_60_ attached. Differently, the C_60_ conjugate with polymer 3 (Figure 3C) shows a spherical morphology with DLS peak position at 140 nm. The size is much bigger than expected. As the AEMA unit number in polymer 3 is 52 and the expected maximum conjugation number of C_60_ is 104 (assuming both amine and amide groups are involved), the expected size should be about 56 nm. It is speculated that C_60_ molecules reacted with two or three polymer chains to form a network structure, resulting in a bigger size with a spherical morphology. C_60_ conjugates with polymers 1–3 displayed inconsistent particle size and morphology, which is not desirable. In polymer 4 containing 35 AEMA units, the above cross‐linking reaction between C_60_ and multiple polymer chains did not occur (Figure 3D). It shows flake‐like morphology with the DLS peak position at 44.1 nm. Unfortunately, after freeze‐drying, the conjugates of C_60_ and polymers 1–4 could not be redispersed in water, forming permanent aggregates. To address this problem, polymer 5 containing 14 repeating units of AEMA was prepared and conjugated with C_60_. The resultant C_60_‐polymer 5 conjugate shows a uniform spherical morphology with DLS peak position at 50.6 nm (Figure 3E). More importantly, after freeze‐drying, the nanoparticles could be redispersed in water, however, with a slightly increased particle size (Figure 3F), indicating a slight aggregation with the DLS peak position shifted to 105.5 nm and also a broader size distribution. It is worth mentioning that although the conjugates of polymers 1–4 and C_60_ could not redisperse into water, their water solutions directly from the dialysis were very stable, with no aggregation observed after several months or longer. Therefore, it is suggested that the C_60_‐polymer conjugates are not to be freeze‐dried after dialysis for further application evaluations.

Among the first set of copolymers synthesized, the copolymer with initial target DPs of 200 (PEGMA)/40 (AEMA) showed promising results as discussed above. Further experiments were conducted in an attempt to tune the particle size of C_60_‐polymer conjugates. In theory, factors such as polymers with larger molecular weight or containing more AEMA repeating units may result in conjugates with more C_60_ molecules incorporated leading to bigger particles. Therefore, polymers 6 and 7 with different target DPs but the same PEGMA:AEMA ratio (100:20 and 300:60, respectively) as polymer 5 (200:40) were designed and synthesized via RAFT polymerization. The results are shown in Table 2. The conversions were also controlled around 50% for PEGMA and polymers 6 and 7 were obtained with DPs of PEGMA/AEMA as 53/6 and 150/22, respectively, determined by the conversion (^1^H NMR).

**TABLE 2:** Synthesis conditions and results of additional poly(PEGMA‐s‐AEMA) copolymers.

Random copolymer	Target DP [PEGMA]/[AEMA]	Conv (mol%)		DP by conversion^a^		*M*_*n*_ (GPC)^b^	PDI (GPC)^b^
		PEGMA	AEMA	PEGMA	AEMA		
6	100/20	53	30	53	6	27,120	1.24
7	300/60	50	37	150	22	59,110	1.30

Abbreviations: AEMA, 2‐aminoethylmethacrylamide; DP, degree of polymerization; GPC, gel permeation chromatography; PDI, polydispersity index; PEGMA, polyethylene glycol methyl ether methacrylate; PMMA, poly(methyl methacrylate).

^a^ DPs were calculated from the conversion: DP = target DP × conv (%).

^b^ PDI and *M*_*n*_ were determined by GPC using PMMA as the calibration standards.

Polymers 6 and 7 were then used for the preparation of C_60_‐polymer conjugates. Their morphologies and DLS results are shown in Figure 3G,H, respectively. The morphology of C_60_‐polymer 6 conjugate is similar to that of C_60_‐polymer 5 conjugate and the DLS peak position is 51.3 nm, also very similar to that of C_60_‐polymer 5 conjugate (50.6 nm). Different from polymers 5 and 6, the conjugate of C_60_‐polymer 7 seems to disperse better than other two polymer conjugates (Figure 3H), which may be due to the increased units of hydrophilic PEGMA. The particle size of C_60_‐polymer 7 conjugate slightly increases with the DLS peak position at 59.5 nm (Figure 3H). The results demonstrate that larger DPs of the polymers do not significantly increase the particle size of the resulting conjugates, indicating that DP may not be a critical factor affecting particle size. The strategy was then changed to adjusting the C_60_ concentration used in the conjugation experiment. Figure 3I,J show the cryo‐TEM images of the polymer 5‐C_60_ (high conc.) conjugate, which was prepared by increasing C_60_ concentration to 30 mg/mL in the conjugation experiment, double the amount previously used. The conjugate shows a spherical morphology and the DLS peak position increases to 69.1 nm. The results demonstrated that increasing C_60_ concentration was more effective than changing DP.

TGA analysis was used to determine the content of C_60_ incorporated in polymers based on the different thermal stability of C_60_ fullerene and polymers under N_2_ atmosphere. It was reported that C_60_ is stable under N_2_ atmosphere up to 650 °C, after which it will partially sublime and form some amorphous carbon residue,^62^, ^63^ while the polymer usually decomposes completely below 500°C. This difference can be used to determine the C_60_ fullerene content in the conjugates.^64^, ^65^ The as‐prepared water solutions of C_60_‐polymer conjugates from dialysis were freeze‐dried for TGA measurements. Figure 3K shows the TGA results of the pristine C_60_, polymer, and C_60_‐polymer conjugates. It can be observed that C_60_ is very stable till 650°C while the as‐prepared polymers decompose completely and lose almost all weight by 454°C (only 2.90% left). The TGA curves of all as‐prepared polymers show similar profiles, thus only one typical polymer curve is shown in Figure 3K for comparison. Surprisingly, the drastic weight loss observed in pristine C_60_ above 800°C was not observed in the C_60_‐polymer conjugates. The C_60_ content of the conjugates was therefore determined by the difference in the remaining weight percentage of the conjugate and the remaining weight percentage of the corresponding pure polymer at 454°C. According to the values determined by TGA measurements, the concentration of C_60_ in the conjugate water solution was determined (Table 3). The concentrations obtained here are for solutions directly from dialysis and could be concentrated further to 10.8 mM with superior stability, which is higher than the reported saturated concentration of C_60_ in toluene (4 mM) and the reported highest concentration of fullerene‐polymer (7.8 mM) in water.^47^ C_60_‐polymer 5 conjugate prepared at a high conjugation concentration got a C_60_ concentration of 4.8 mM (Table 3). The solution was very stable after more than 1 year of storage. For its stability and uniform morphology, it was chosen for biological evaluation as discussed below.

**TABLE 3:** C_60_ content in conjugates (wt%) and solution concentration of C_60_‐polymer conjugates (right after dialysis) based on C_60_.

Random copolymer + C_60_	C_60_ content from TGA measurements (wt%)	C_60_ water solution concentration (mg/mL)	C_60_ water solution concentration (mM)
Polymer 1 + C_60_	53.1	1.48	2.1
Polymer 2 + C_60_	50.2	2.34	3.3
Polymer 3 + C_60_	49.1	1.50	2.1
Polymer 4 + C_60_	43.4	1.35	1.9
Polymer 5 + C_60_	33.6	1.50	2.1
Polymer 6 + C_60_	35.6	0.85	1.2
Polymer 7 + C_60_	35.4	1.21	1.7
Polymer 5 + C_60_ (high conjugation conc.)	34.7	3.48	4.8

Abbreviation: TGA, thermal gravimetric analysis.

### 3.4 Phototoxicity to A549 cells under light irradiation

**FIGURE 4:**
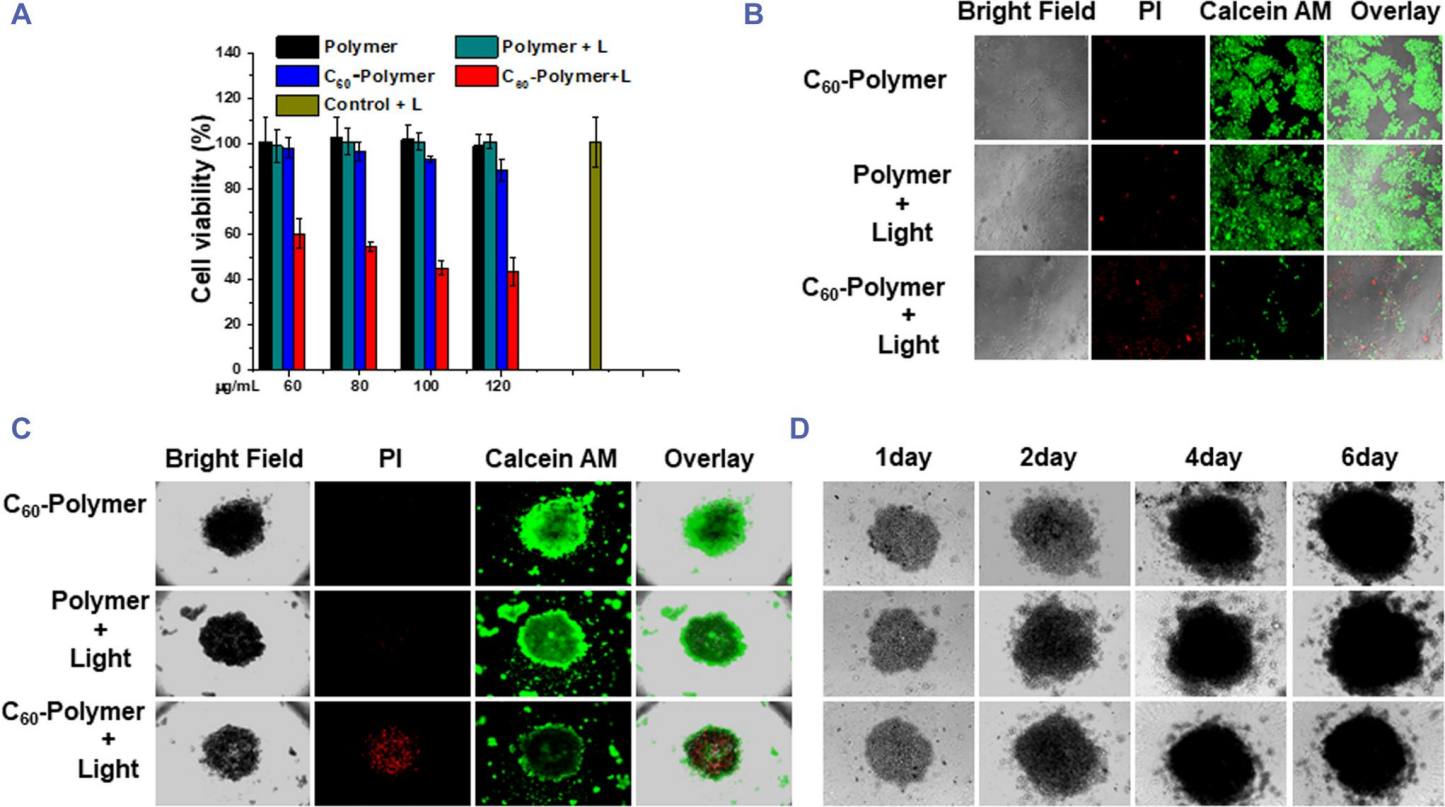
Phototoxicity to A549 cells. (A) MTT assay results. A549 cells were treated with C_60_‐polymer conjugate of different concentrations (60, 80, 100, 120 μg/mL) and polymer alone, with and without light irradiation (450 nm, 12 J cm^−2^, 5 min). (B) Calcein AM/PI staining assay results of 2D A549 cells. 2D A549 cells were treated with C_60_‐polymer conjugate or polymer (100 μg/mL), with or without light irradiation (450 nm, 12 J cm^−2^, 5 min). (C) Calcein AM/PI staining assay results of A549 MCTSs. A549 MCTSs were treated with C_60_‐polymer conjugate or polymer (100 μg/mL), with or without light irradiation (450 nm, 12 J cm^−2^, 5 min). (D) Diameter changes of A549 MCTSs after being treated with C_60_‐polymer conjugate or polymer (100 μg/mL), with or without light by increasing days. AM/PI, calcein am propidium Iodide; MCTSs, multicellular tumor spheroids; MTT, 3‐(4,5‐dimethylthiazol‐2‐yl)‐2,5‐diphenyltetrazolium bromide.

Fullerenes have been explored in PDT therapy.^66^ In order to investigate the PDT effect of C_60_‐polymer conjugate, cells were incubated with the C_60_‐polymer conjugate or polymer (as a comparison) at different concentrations for 4 h and then were irradiated for 5 min (450 nm, 12 J cm^−2^). MTT assay was performed to determine the relative viability of the cells. A549 cells of control group exhibited no loss of viability after being irradiated for 5 min (450 nm, 12 J cm^−2^), which indicated that the control cells remained safe under irradiation. Without light irradiation, C_60_‐polymer conjugate or polymer exhibited negligible dark toxicity to A549 cells (Figure 4A). Interestingly, the phototoxicity of C_60_‐polymer conjugate was remarkable when irradiated by light. The PDT effect of the C_60_‐polymer conjugate was also detected by calcein AM/PI staining assay (Figure 4B). Live cells can convert the nonfluorescent cell‐permeant Calein AM into the green fluorescent calcine in the presence of intracellular esterase, and dead cells could be stained by red fluorescent with PI. A549 cells treated with the C_60_‐polymer conjugate followed by light irradiation died (as represented by the significant reduction of green fluorescence and increase of red fluorescence). Cells treated with polymer with light irradiation showed stronger green fluorescence, as well as the cells in the group of C_60_‐polymer conjugate without light irradiation. The results indicated that C_60_‐polymer conjugate could destroy cancer cells in the light irradiation.

The calcein AM/PI staining assay was performed to determine the viability of MCTSs.^13^ As expected, the cells of the MCTSs treated with C_60_‐polymer conjugate without light or treated with polymer with light irradiation were all alive, which showed strong green fluorescence. However, the strong red fluorescence was observed in MCTSs treated with C_60_‐polymer conjugate with light irradiation and the green fluorescence in these MCTSs could hardly be detected (Figure 4C).

In addition, we observed the phototoxicity effect on the kinetics of 3D tumor growth. After treatment with C_60_‐polymer conjugate without light or treated with polymer with light, the size of MCTSs was still growing rapidly. However, the diameter of MCTSs treated with C_60_‐polymer conjugate with light stopped changing and became much smaller than that of other two groups after 6 days, indicating that the C_60_‐polymer conjugate had strong phototoxicity against A549 MCTSs (Figure 4D).

### 3.5 Light‐induced ROS generation in vitro

**FIGURE 5:**
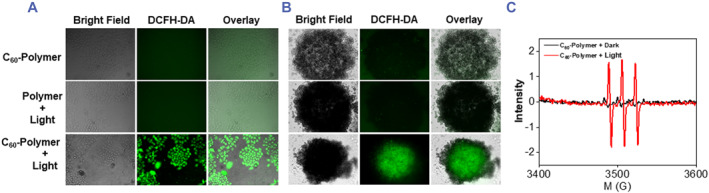
Light‐induced ROS generation in vitro. (A) 2D A549 cells and (B) A549 MCTSs were incubated with the DCFH‐DA (10 μM) in the absence or in the presence of light. The samples of the light group were irradiated for 5 min (450 nm, 12 J cm^−2^). (C) EPR signals of ^1^O_2_ without or with light irradiation (450 nm, 12 J cm^−2^, 5 min) of C_60_‐polymer conjugate in the presence of TEMP. DCFH‐DA, 2′,7′‐dichlorofluorescein diacetate; EPR, electron paramagnetic resonance; MCTSs, multicellular tumor spheroids; ROS, reactive oxygen species; TEMP, 2,2',6,6'‐tetramethylpiperidine.

The ability of C_60_‐polymer conjugate to generate ROS within A549 cells was detected using the 2ʹ,7ʹ‐dichlorodihydrofluorescein diacetate (DCFH‐DA) fluorescent probe. DCFH‐DA is a nonfluorescent compound that becomes highly fluorescent 2ʹ,7ʹ‐dichlorofluorescein (DCF) on oxidation by intracellular ROS such as ^1^O_2_.^67^ As illustrated in Figure 5A, after light irradiation, strong green intracellular fluorescence of C_60_‐polymer conjugate‐treated cells was observed, indicating the generation of ROS in cells. However, no green fluorescence was observed in the cells treated with C_60_‐polymer with no light irradiation and those treated with polymer with light irradiation.

In addition, the abilities of these compounds to generate ROS in A549 MCTSs were also detected. As shown in Figure 5B, the A549 MCTSs treated with C_60_‐polymer conjugate exhibited strong green intracellular fluorescence after light irradiation while no green fluorescence was observed in the A549 MCTSs of other two groups, which were consistent with that in A549 2D cells. These results verified that the C_60_‐polymer conjugate could produce ROS in A549 cells after light irradiation. ^1^O_2_ production is the crucial factor in the phototoxicity of A549 cells induced by C_60_‐polymer conjugate.

Furthermore, it has been reported that ^1^O_2_ plays an important role in the phototoxicity of many functionalized fullerenes.^68^ To determine whether the as‐prepared C_60_‐polymer conjugate could generate ^1^O_2_ under light irradiation, we further employed EPR spectroscopy to detect ^1^O_2_ generation. As shown in Figure 5C, after being irradiated, a characteristic ^1^O_2_‐induced signal, 2,2,6,6‐tetramethylpiperidine‐1‐oxyl of C_60_‐polymer conjugate was observed. However, no ^1^O_2_ signal was observed when C_60_‐polymer conjugate was in the dark.

## 4 DISCUSSION

The poor solubility of fullerenes severely limits its potential biomedical applications. In order to prepare highly water‐dispersible C_60_ fullerene nanoparticles, a series of random copolymers with a fixed number of PEGMA units (around 100) and different number of AEMA units were successfully designed and synthesized by RAFT polymerization. RAFT is a well‐known technique to prepare polymer materials with well control of the polymerization process, polydispersity of molecular weight, and structure of polymers. The side chains of polymers from AEMA units can conjugate with C_60_ fullerene molecules via amine addition and the PEGMA units nearby C_60_ molecules can wrap them and create a hydrophilic microenvironment, resulting in highly water‐dispersible C_60_ nanoparticles with excellent stability. Various instrumental characterization proved the intact structure of C_60_ after conjugation so that C_60_ keeps photodynamic property. By optimizing the experiments, water solutions containing uniform, spherical C_60_‐polymer nanoparticles with particle size of 50–70 nm were obtained. C_60_ concentration of these solutions is very high, which can be further concentrated to a concentration of 10.8 mM, much higher than the saturated concentration of C_60_ in toluene (4 mM) and the reported highest concentration of fullerene‐polymer (7.8 mM) in water. More importantly, the solution is very stable during more than 1 year's storage period. Compared with the previous studies of making water‐dispersible fullerenes, the strategy developed in this study is simple, efficient, and scalable, which may potentially impact the future biomedical applications of fullerenes.

## 5 CONCLUSIONS

The C_60_‐polymer conjugate showed strong cytotoxicity to A549 cells under light irradiation. Notably, C_60_‐polymer conjugate did not show any appreciable cytotoxicity under dark conditions, which further demonstrated that C_60_‐polymer conjugate had an attractive targeting and safety profile. In addition, MCTSs have been gradually accepted as a valid 3D cancer model to imitate solid tumors in vivo. The C_60_‐polymer conjugate demonstrated efficient PDT effect in A549 MCTSs under irradiation. These results indicated that C_60_‐polymer conjugate may be suitable for the PDT of deep cancer tissue.

## AUTHOR CONTRIBUTIONS

**Dan Wang**: Conceptualization; methodology; investigation; data collection and analysis; funding acquisition; writing – original draft. **Jianyang Zhao**: Investigation; data collection and analysis; writing – review & editing. **Roger J. Mulder**: Investigation; data collection and analysis; writing – review & editing. **Julian Ratcliffe**: Investigation; data collection and analysis. **Chunru Wang**: Validation; conceptualization; supervision. **Bo Wu**: Investigation; data collection and analysis; writing – review & editing. **Jinquan Wang**: Conceptualization; methodology; investigation; data collection and analysis; writing – review & editing. **Xiaojuan Hao**: Methodology; conceptualization; validation; visualization; funding acquisition; supervision; writing – review & editing.

## CONFLICT OF INTEREST STATEMENT

The authors declare that there are no competing interests.

## ETHICS STATEMENT

There are no experiments dealing with animal, human subjects or tissue samples from human subjects in this study.

## Data Availability

No data were used in the research described in this article.
